# Dr. Harris and the New Orleans Journal of Medicine

**Published:** 1847-06

**Authors:** 


### DR. HARRIS AND THE NEW ORLEANS JOURNAL OF MEDICINE.

Dr. Harris has written to us complaining that the editors of the New Orleans Medical and Surgical Journal, in the May number, have done him injustice in their strictures on his paper entitled, "*Pathology and Treatment of Fever.*" We have not received that number of the New Orleans Journal, and are not informed as to the extent or character of the strictures, except as adverted to in the reply of Dr. Harris. What he particularly complains of is, that the editors accuse him of " inexperience and want of candor." We can undertake to say, if such charges have been preferred against Dr. Harris, that they are unjust. Dr. H. has been engaged in the practice of medicine for more than a dozen years, and in that time has seen much of those diseases which abound in southern latitudes. In respect to candor, no one who has known him ever questioned that, however much he may be mistaken in his views, he is always honest in the entertainment and expression of them. For the rest, differences of opinion among physicians, both as to pathology and modes of practice, must prevail, and every writer who submits his thoughts to the profession may make up his mind to have them fully and freely canvassed. We cannot for a moment entertain a suspicion that the able and impartial editors of the New Orleans Medical Journal would willingly do Dr. Harris injustice, and therefore decline publishing his reply to their notice of his paper.

